# Antimicrobial Sol–Gel Glassy Surfaces for Modification of Dental Implant Abutments to Reduce Microbial Adhesion

**DOI:** 10.3390/gels11110882

**Published:** 2025-11-03

**Authors:** Özlem Çölgeçen, Murat Akarsu, Esin Akarsu, Ataç Uzel, Feyzan Özdal Kurt, Eyüp Sabri Topal, Gül Merve Gençer, Ahmet Keski, Emre Yavuz

**Affiliations:** 1 Department of Prosthodontics, Faculty of Dentistry, İzmir Katip Çlebi University, 35640 Izmir, Türkiye; ahmetkeski@gmail.com; 2 Department of Chemistry, Faculty of Science, Akdeniz University, 07058 Antalya, Türkiye; akarsu@akdeniz.edu.tr (M.A.); esinakarsu@akdeniz.edu.tr (E.A.); gulmerve.gencer@antalya.edu.tr (G.M.G.); emreyavuz08@gmail.com (E.Y.); 3 Department of Biology, Basic and Industrial Microbiology Section, Faculty of Science, Ege University, 35100 Izmir, Türkiye; atac.uzel@ege.edu.tr; 4 Department of Molecular Biology, Faculty of Engineering and Natural Sciences, Manisa Celal Bayar University, 45140 Manisa, Türkiye; feyzanozdalkurt@yahoo.com; 5 Department of Mechanical Engineering, Faculty of Engineering, Akdeniz University, 07058 Antalya, Türkiye; estopal@akdeniz.edu.tr

**Keywords:** dental implant, antimicrobial coatings, abutment, sol–gel, Ti6Al4V, glassy coatings, bacterial adhesion, biocompatibility, surface modification

## Abstract

Microbial colonization is a major factor contributing to peri-implantitis, and creating durable glassy surfaces with antimicrobial agents such as silver and copper may reduce microbial accumulation on dental abutments. This study aimed to develop antimicrobial thin-film glassy surfaces on Ti6Al4V alloy and to evaluate their surface and mechanical properties, antimicrobial effectiveness, and biocompatibility before and after thermal aging. A sol–gel-derived glassy matrix (G) was synthesized, and two antimicrobial coatings were prepared by incorporating ionic Ag (GAg) or a combination of Ag/Cu (GAgCu). Ti6Al4V specimens; these were either left uncoated or dip-coated with G, GAg, or GAgCu and cured at 450 °C. Half of the specimens underwent thermal aging between 5 °C and 55 °C for 3000 cycles. Surface roughness, contact angle, hardness, adhesion strength, scratch resistance, cytotoxicity (Agar diffusion and MTT assay on L929 fibroblasts), and microbial adhesion were evaluated using *Streptococcus sanguinis*, *Porphyromonas gingivalis*, and *Candida albicans* as representative oral microorganisms. Both coatings exhibited low surface roughness, hydrophilic surfaces, improved hardness, and significantly reduced microbial adhesion for all tested species. GAg showed superior mechanical properties, whereas GAgCu demonstrated a relatively stronger antimicrobial effect. Cytotoxicity tests indicated that all coatings were biocompatible at levels suitable for oral use. Overall, these coatings demonstrated strong adhesion, durability, and antimicrobial activity, suggesting their suitability for dental abutments made of Ti6Al4V.

## 1. Introduction

Titanium-based materials, especially Titanium–Aluminum–Vanadium (Ti6Al4V) alloy, are key biomaterials used in implant treatment because of their good biocompatibility and superior mechanical properties [1]. However, a number of in vitro [2,3] and in vivo studies [4,5] have reported that the surfaces of titanium-based implant parts are susceptible to microbial adherence under biological conditions, leading to microbial colonization and biofilm formation. There is a strong correlation between the number of microorganisms adhering to the surface and the incidence of peri-implant infections [6]. Infections in the peri-implant area caused by microbial accumulation on the implant components are a significant problem, affecting both the postoperative healing [7] and the long-term success of implant-supported restorations [8]. In the transmucosal region of prosthetic or healing abutments, microbial adhesion and the subsequent formation of biofilms represent the critical initial steps in the pathogenesis of peri-implant diseases.

In dental implantology, healing and/or prosthetic abutments serve as a critical transition zone between the osseointegrated implant embedded in bone and the oral environment. These abutments are in contact with the gingival tissue, and some parts are directly exposed to saliva and the oral microbiota [9,10]. Healing abutments are temporarily placed following implant surgery and left in position throughout osseointegration to promote soft tissue healing and to establish an appropriate emergence profile. Their surface characteristics, especially roughness and wettability, play a decisive role in early biofilm formation and may increase the susceptibility to peri-implant inflammation. In contrast, prosthetic abutments serve as long-term supports for the final restoration and remain in contact with both soft tissues and the oral cavity. Consequently, their design and surface properties are crucial for maintaining peri-implant health and preventing microbial colonization [11,12]. Therefore, the development of surfaces capable of reducing microbial adhesion is of paramount importance in implant dentistry. In this regard, surface engineering strategies have gained increasing attention as a means to both minimize bacterial colonization and maintain biological integration with surrounding tissues.

One of the strategies to prevent biofilm formation on implants is to coat the surface with antibacterial thin films. Many approaches have been proposed for this purpose, such as coatings with silver (Ag) using the physical vapor deposition technique [13], Ag ion implantation [14,15], electrophoretic deposition of antibiotic-loaded microsphere–alginate particles [16], thermal-sprayed Ag ions [17], anodic oxidation of the titanium surface [18], and gradient Ag-titanium oxide thin-film using reactive sputtering [19]. It is well known that these methods are expensive and require special equipment and line-of-sight techniques, by which coating the complex-shaped structures is extremely difficult. On the other hand, the solutions obtained by the sol–gel method can be applied by one of the wet coatings, such as dipping, spraying, etc., to obtain homogeneous coatings [20]. Besides being simple, applicable, and economical, the sol–gel method allows the manipulation of the structure of the final material at the nano-level by the use of molecular precursors and their controlled reactions of hydrolysis and condensation [21]. Silicate-based materials and coatings with high purity for biomedical purposes can be produced by the hydrolysis and condensation of highly purified alkoxysilanes as a precursor. Hereby, the formation of the glass phase of silicate materials takes place at much lower temperatures compared to the traditional melting method, which leads to better adhesion of the coating to the surface due to lower thermal stress caused on the metal substrate [22,23].

In the literature, silicate-based coatings incorporated with micron-sized particles of silver phosphate or silver-zeolite are produced by the sol–gel method on the sheets of stainless steel and aluminum substrates [24]. Only the initial adhesion and wear resistance of silicate coatings have been characterized, while their post-aging surface properties have not been thoroughly examined. Moreover, these coatings have not been subjected to advanced intraoral tests, such as thermal aging, which are essential for clinical evaluation. The applicability of a novel coating in the oral environment largely depends on its durability under these conditions. For abutment coatings, strong adhesion to the titanium substrate and resistance to intraoral thermal fluctuations are critical. If the coating fails to form a durable bond and becomes prone to detachment under the combined influence of hydrolytic stress and repeated temperature changes during aging, its antimicrobial effectiveness becomes clinically irrelevant. The naturally moist oral environment, along with frequent exposure to beverages, promotes recurrent hydrolytic stress, while the consumption of hot and cold drinks further amplifies thermal cycling. These combined factors generate interfacial stresses that can weaken adhesion [25,26] and adversely affect essential surface properties, including hardness and roughness [27]. Therefore, thermal cycling tests provide a valuable model for evaluating the long-term stability of functional surface characteristics. Consequently, developing biocompatible abutment coatings that maintain both antimicrobial efficacy and resistance to intraoral thermal changes represents a critical research priority.

Silver and copper ions exhibit strong antimicrobial effects by both inhibiting biofilm formation and disrupting existing biofilms. They compromise the integrity of microbial membranes [28,29,30], increase permeability, and generate reactive oxygen species that damage DNA, proteins, and lipids [28,31,32]. Additionally, they inactivate bacterial enzymes, disrupt metabolic pathways, and bind to DNA, thereby interfering with replication and transcription [28,29,33]. As a result, biofilm formation is significantly reduced by either killing microorganisms or inhibiting their proliferation. Moreover, since these ions have been reported to be non-toxic to human cells at low doses [34], they are generally preferred in the fabrication of biomaterials or in surface modifications.

The aim of this study was to develop sol–gel-derived glassy coatings incorporated with silver alone and silver–copper combinations in ionic form on Ti6Al4V alloy for dental purposes. The biomechanical and surface characteristics of the studied coatings were extensively investigated to obtain a suitable film candidate for long-term oral use in clinics. The null hypothesis of this study was that sol–gel silicate coatings containing antimicrobial additives would have no significant effect on microbial adhesion, surface properties, or biocompatibility.

## 2. Materials and Methods

### 2.1. Specimen Preparation

A total of 491 square-shaped specimens (3 mm thickness, 10 × 10 mm size) were prepared using guillotine cutting, from Grade 5-ELI medical titanium alloy sheet (Ti6Al4V-Shanghai Huaxia Industry ASTM-F136 [35], ELI, Ti6AL4V, ISO 5832-3 [36], Lot No: 1309-H0211). A single surface of the specimens was machined using a computer numerical control milling machine (Taksan TMC500V, Taksan Incorporated Company, Kayseri, Turkiye) at a 200 m/min cutting speed with 0.1 mm cutting depth. After that, the roughness of all specimens was measured using a profilometer (Surftest-310, Mitutoyo, Japan) to verify whether the roughness average (Ra) was below 0.2 µm, which is the threshold roughness value for dental applications determined by Quirynen and Bollen [37]. All specimens were divided into four groups. These were:

- **Ti6Al4V**: Uncoated control group;
- **G**: Coated group with only glassy coating;
- **GAg:** Coated group with a mixture of the G+ 2% Ag;
- **GAgCu**: Coated group with a mixture of the G+ 1% Ag+ 1% Cu.

### 2.2. Synthesis and Application of the Glassy Coatings

#### 2.2.1. Preparation of the Glassy Matrix Solution

**Figure 1:**
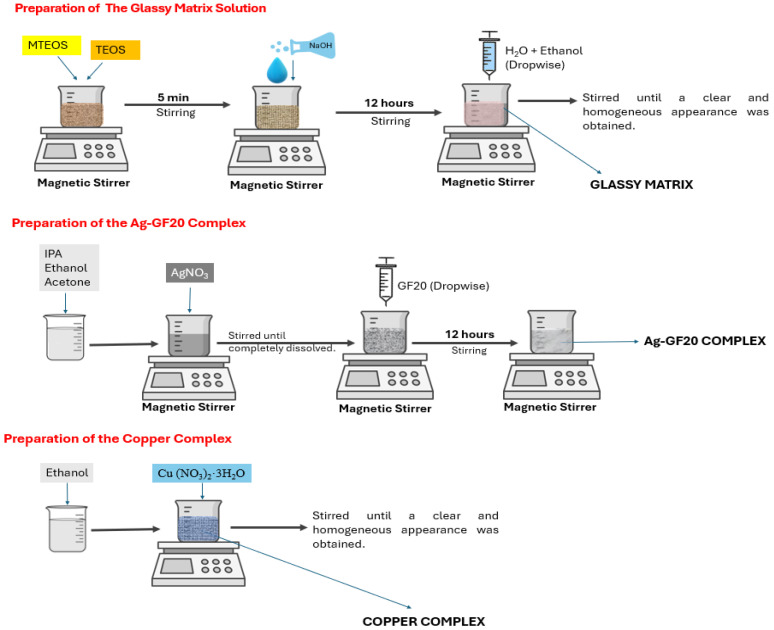
Preparation steps and synthesis route of the glassy matrix, Ag-GF20, and copper complexes.

The synthesis route of the glassy matrix solution is presented in Figure 1.

In the first step of the synthesis route of the glassy matrix, 75.243 g (0.422 mol) Methyltriethoxy silane (MTEOS, 99%,Sigma-Aldrich, St. Louis, MO, USA) and 22.048 g (0.106 mol) tetraethyl orthosilicate (TEOS, >99%, Sigma-Aldrich, St. Louis, MO, USA) were weighed in a bottle and stirred for 5 min at room temperature. A 2.72 g (0.068 mol) amount of sodium hydroxide (NaOH, ≥98%, Merck, Darmstadt, Germany) was added to this mixture and stirred for 12 h at room temperature to produce a clear solution. A solution comprising 8.52 g (0.473 mol) of H_2_O and 120 g of ethyl alcohol (≥99.8%, Sigma-Aldrich, St. Louis, MO, USA) was added dropwise to 78.88 g of silane-NaOH solution and stirred for two hours.

#### 2.2.2. Preparation of the Silver-GF20 Complex and the Copper Complex

The synthesis routes of these complexes are also presented in Figure 1. Firstly, 5.51 g silver nitrate (AgNO_3_, ≥99%, Sigma-Aldrich, St. Louis, MO, USA) was dissolved in a solvent mixture of 60.30 g isopropyl alcohol (≥98%, Sigma-Aldrich, St. Louis, MO, USA), 116.07 g ethyl alcohol (≥99.8%, Sigma-Aldrich, St. Louis, MO, USA) and 12.92 g acetone (≥99.9%, Sigma-Aldrich, St. Louis, MO, USA), and then 5.21 g 3-triethoxysilyl propyl succinic anhydride (GF20, 95%, Gelest Inc., Morrisville, PA, USA) was dropped slowly into it. This solution of Ag-GF20 complex was stirred for twelve hours at room temperature. For preparing the copper complex, 6.69 g copper (II) nitrate trihydrate (Cu (NO_3_)_2_·3H_2_O, ≥99%, Sigma-Aldrich, St. Louis, Missouri, USA) was dissolved in 93.31 g ethyl alcohol in a bottle. The metallic copper content of the solution was 1.75 percent by weight.

#### 2.2.3. Preparation of the Coating Solutions

**Figure 2:**
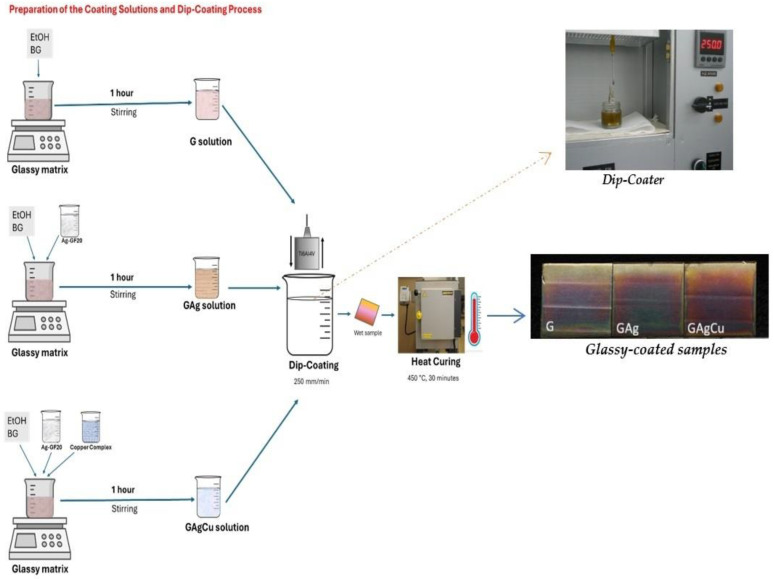
Preparation of the glassy coating solutions and application of the dip-coating process. After coating, the samples were cured by heat in a furnace at 450 °C for 30 min.

The compositions of the prepared coating solutions are given in Table 1, and the laboratory steps are summarized in Figure 2. Each component in Table 1 was dropped slowly under stirring in the given order under continuous stirring for an additional hour at room temperature to obtain the final coating solutions. Each of them had a solid content of 7.5 percent by weight. The preliminary optimization trials for antimicrobial coating combinations are presented in Supplementary Materials.

#### 2.2.4. Dip-Coating Process

The substrates were cleaned with an ultrasonic bath with a 5% aqueous detergent solution (P3 Almeco 18, Henkel, Germany) at 80 °C for 5 min. Then, the substrates were rinsed with distilled water and placed in a 10% aqueous nitric acid solution for 5 min for neutralization. Specimens were rinsed with distilled water, dried at 100 °C for one hour, and cooled to room temperature. After cleaning, the Ti6Al4V substrates were coated using the dip-coating method at a constant withdrawal speed of 250 mm/min with the respective coating solutions. The coating process was employed with a laboratory-scale, custom-made, programmable dip-coater capable of maintaining both withdrawal speed and dwell time (Figure 2). The substrates were positioned vertically during the process. Subsequently, the wet substrates were heated to 450 °C for one hour and then cured in a furnace (LH 60/12, Nabertherm, Germany) at that temperature for 30 min. They were then allowed to cool naturally to room temperature. Finally, the coated specimens were obtained as pure glassy (G), glassy containing 2% silver (GAg), and glassy containing 1% silver and 1% copper (GAgCu).

### 2.3. Thermal Aging Process

The stability of bonded dental surfaces can be evaluated by thermal aging procedures that replicate the oral environment [38]. In the present investigation, intraoral thermal fluctuations were simulated by subjecting half of the specimens from each group to 3000 cycles in a thermocycling device (Thermocycle tester, Gökçeler Machine, Istanbul, Turkiye). During cycling, the samples were alternately transferred between water baths at 5 °C and 55 °C, with a dwell time of 30 s in each water bath.

### 2.4. Mechanical and Surface Characteristics of the Coatings

#### 2.4.1. Measurement of the Adhesion Strength

The adhesion strength of the new glassy surface to Ti6Al4V was measured via two different methods. The first test was performed in accordance with the American Society for Testing and Materials (ASTM) standard D3359 [39]. In this test, a multi-blade Erichsen Multi-cross Hatch Cutter model 295 was used. After scratching the coating surface with this tool, a standardized band was stuck to the surface and then pulled away. The amount of peeled-off film, together with the band, was examined, and the adhesion level of the coating layer was graded according to the given standards, ranging from 0B to 5B (5B: excellent adhesion, cuts have smooth edges with no coating detachment; 4B: small flakes detach at intersections, ≤5% area affected; 3B: coating flakes along edges and partly at intersections, 5–15% area affected; 2B: coating detaches in ribbons along cuts or intersections, 15–35% area affected; 1B: flaking along edges and large ribbons, 35–65% area affected; 0B: severe detachment exceeding 65%, poor adhesion) [39].

A progressive scratch test was performed using a CSM Micro Indenter (CSM Instruments, Needham, MA, USA) in a linear scratch configuration to determine the film layer’s adhesion strength. This method, which utilizes a diamond indenter with a 100 µm tip radius, involves linearly increasing the load from 0.05 N to 10 N. The primary indicator for the coatings’ adhesion strength was the second critical load value (Lc2), which marks the point of complete coating breakthrough. For each group, five critical load values were meticulously determined by analyzing both acoustic emission signals and optical microscope data, providing crucial insights into the coating’s mechanical stability and its bonding quality to the substrate.

#### 2.4.2. Measurement of the Contact Angle

Contact angle analysis was carried out with a goniometer (Ramé-Hart 100-FO, Netcong, NJ, USA) using the sessile drop technique. For all measurements, distilled water was selected as the testing liquid, and the droplet volume was adjusted to 50 µL. The droplets were gently placed from a distance of 1 cm above the specimen surface and allowed to stabilize for 3 s. The angle formed by the droplet on the surface was then evaluated from its mirror image with the aid of a protractor.

#### 2.4.3. Measurement of Surface Roughness

Surface roughness values were measured with a profilometer (Surftest-310, Mitutoyo, Japan) using the measurement parameters, including a length of 4 mm, a probe speed of 0.5 mm/s, and a cut-off value of 0.25 mm. Five different measurements were performed on the surface of each specimen, and the Ra values were recorded. The profilometer was calibrated after every ten measurements.

#### 2.4.4. Measurement of Surface Hardness

The thin thickness of the coated films required the use of a Nano-Hardness Tester (CSEM Instruments, Neuchâtel, Switzerland) for surface hardness evaluation. A diamond conical indenter with a maximum load of 0.5 mN was employed, and at least three measurements were performed on each specimen surface. For comparison, the hardness of the uncoated Ti6Al4V alloy was measured using a Micro-Hardness Tester (Shimadzu HMV-2, Kyoto, Japan) under a 50 g load, applied for 20 s, and the results are reported as Vickers hardness values.

### 2.5. Microbial Adhesion Tests

Before microbial adhesion tests, a pellicle layer was formed on all specimen surfaces by exposing them to artificial saliva containing mucin (Type II; Sigma-Aldrich, St. Louis, MO, USA). The saliva solution was prepared according to the Fusayama formulation, which is considered to best simulate the composition of natural saliva. For sterilization, the prepared solution was filtered through single-use syringe filters with a pore size of 0.45 μm. The specimens were subjected to this solution for 2 h at 37 °C, rinsed with saline for 30 s, and air-dried for 1 h in sterile Petri dishes.

#### Microbial Assay and Fluorescence Analysis

Microbial adhesion measurements were conducted following the method described by Buergers et al. [40]. Three different microbial strains (*Streptococcus sanguinis* ATCC 10556, *Porphyromonas gingivalis* ATCC 33277, and *Candida albicans* DSM 5817) were used. Each strain was cultured for 24 h in its respective medium: Brain Heart Infusion Broth (Oxoid, Basingstoke, UK), GAM Broth supplemented with hemin (5 µg/mL) and menadione (1 µg/mL), and Yeast Peptone Dextrose Broth (YPD, Merck, Darmstadt, Germany). Microbial cells were harvested by centrifugation at 291 RCF for 5 min at 18 °C, washed twice, and resuspended in the same buffer. Cell suspensions were adjusted to an optical density of 0.3 at 540 nm.

**Figure 3:**
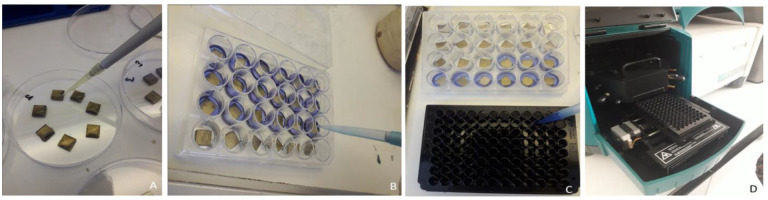
Microbial adhesion test procedure. (**A**) A microbial suspension was pipetted onto each specimen, and (**B**) resazurin solution was added to each well. (**C**) The resulting resorufin solutions were transferred to an opaque black 96-well microplate, and (**D**) absorbance was measured using a spectrophotometer.

For adhesion testing, 50 µL of microbial suspension was pipetted onto each specimen surface (Figure 3A), placed in sterile Petri dishes, and incubated for 2.5 h at 37 °C. Non-adherent cells were removed by gently washing specimens twice with 500 µL phosphate-buffered saline, after which they were transferred into 24-well plates. Each well received 350 µL of resazurin solution (Figure 3B) and was incubated for 2 h at 37 °C to allow the reduction of resazurin to resorufin. The reaction was then stopped and stabilized with 3% (*w*/*v*) sodium dodecyl sulfate. The resulting resorufin solutions were transferred to a 96-well plate (Figure 3C), and fluorescence intensity was measured with a spectrophotometer (excitation: 530 nm, emission: 570 nm; Varian) (Figure 3D). Fluorescence values were used to quantify the extent of microbial adhesion.

### 2.6. Cytotoxicity Tests

To evaluate the suitability of the novel glassy coatings as a dental material, commonly used restorative materials such as composite and amalgam fillings that are located in proximity to the gingiva were included as control groups in the cytotoxicity tests. Composite specimens (Cavex Quadrant Universal LC, Cavex, Haarlem, The Netherlands) were fabricated using a 10 × 10 × 2 mm mold, pressed with a glass slide, and polymerized following the manufacturer’s recommendations. The glossy surfaces that had been in contact with the glass slide were used as the test surface. To prepare amalgam specimens, pre-capsulated amalgam (Cavex Non-Gamma-2, Cavex, Haarlem, The Netherlands) was mixed in an amalgamator (Rotomix, 3M Espe, Seefeld, Germany) and packed into the mold using an amalgam plugger, then compressed with a glass slide. After 24 h, the specimens were removed from the mold, and the surface adjacent to the glass slide was polished to a mirror-like finish with 1200-grit sandpaper and a burnisher.

Cytotoxicity of the glassy coatings was evaluated using two distinct methods. The agar diffusion test, performed only on non-aged specimens, served as a preliminary assessment to identify any cytotoxic effects and to provide visual microscopic evidence. In contrast, the MTT (3-[4,5-dimethylthiazol-2-yl]-2,5 diphenyl tetrazolium bromide) assay is based on the conversion of MTT into formazan crystals by living cells, which determines mitochondrial activity. It was conducted on both aged and non-aged specimens to quantify cell viability and proliferation. Autoclave sterilization was applied to specimens at 121 °C for 15 min before testing to prevent contamination. Experiments utilized the mouse fibroblast cell line (L929) at passages 8–10.

#### 2.6.1. Cell Cultivation

L929 cells were cultured in DMEM/F-12 medium supplemented with 10% fetal bovine serum, 1% L-glutamine, and 1% penicillin–streptomycin. The L929 cell line was bought from American Type Culture Collection (ATCC, Manassas, VA, USA). Cultures were maintained at 37 °C in a humidified incubator with 5% CO_2_ (Hera-Cell, Kendro Laboratory, Germany). The medium was replaced every two days, and cell morphology was monitored using a phase-contrast inverted microscope (IX71, Olympus, Tokyo, Japan). For agar diffusion and MTT assays, cells were harvested from confluent cultures, subcultured using 0.25% trypsin-EDTA, and seeded at 1.5 × 10^5^ cells/mL per well in tissue culture plates [41].

#### 2.6.2. Agar Diffusion Test

ISO 10993-5 [42] was followed to perform the agar diffusion test. L929 cells were grown in 6-well tissue culture plates until reaching 85–90% confluence. The culture medium was then replaced with a 1:1 mixture of nutrient medium and 1% agar, which solidified to form an agar layer. Sterilized specimens’ coated or polished surfaces were carefully placed on the solidified agar, and a cell control group without any material was included. Plates were incubated for 72 h at 37 °C under 5% CO_2_. Following incubation, specimens and agar were removed, and the cells were stained with crystal violet. Cell morphology was examined under an inverted phase-contrast microscope (CK40-F200, Olympus, Japan) to detect cytotoxic effects in cells beneath or adjacent to the specimens. Cytotoxicity was graded according to ISO 10993-5 [42], based on morphological changes, cell lysis, and percentage of viable cells: grade 0, no cytotoxic effect with normal morphology; grade +1, slight cytotoxicity with minimal lysis and >90% viability; grade +2, mild cytotoxicity with light lysis and 70–90% viability; grade +3, moderate cytotoxicity with significant lysis and 50–70% viability; and grade +4, severe cytotoxicity with extensive lysis and <50% viability.

#### 2.6.3. MTT Assay

Both non-aged and aged specimens were placed into sterile 24-well tissue culture plates. The L929 cells in growth medium were added to each well and incubated at 37 °C in a humidified atmosphere containing 5% CO_2_ for 48 h. At the end of the incubation, the cultures were processed for the MTT assay. Cells were exposed to 0.5 mg/mL MTT for the final 4 h, after which the medium was removed. The resulting formazan crystals were dissolved in dimethyl sulfoxide (DMSO), and the absorbance was measured immediately using a microplate reader at 570 nm with a 690 nm reference filter (VersaMax, Molecular Devices, San Jose, CA, USA). Cell proliferation was quantified via the MTT assay three times at two-day intervals throughout the culture period [43].

### 2.7. Statistical Analyses

Statistical analyses were conducted using SPSS Statistics V.29 (IBM, New York, NY, USA) for Windows. Data normality was evaluated using the Shapiro–Wilk test and Q-Q plots, while homogeneity of variances was assessed with Levene’s test. Two-way ANOVA was used for the evaluation of differences between groups for surface roughness, contact angle, surface hardness, scratch resistance, and cytotoxicity (factors: Material and Thermal Aging). Three-way ANOVA was used within general linear models to evaluate microbial adhesion test results (factors: Material, Thermal Aging, and Microbial Strain). Post hoc comparisons were performed using the Bonferroni test with a significance level of 0.05 and 95% confidence intervals.

## 3. Results

### 3.1. Surface Morphology and Elemental Analysis

**Figure 4:**
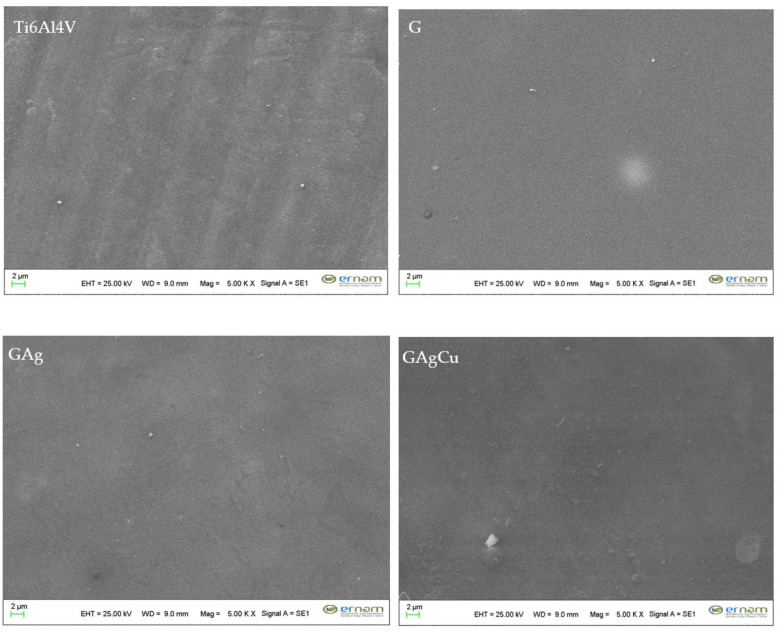
Surface topographic images of the experimental groups obtained by SEM analyses after 3000 thermocycles at ×5000 magnification. No defects or delamination on the surfaces were observed.

**Figure 5:**
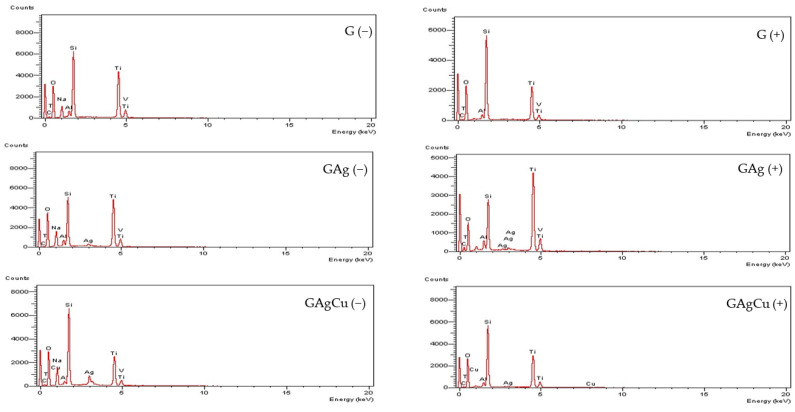
EDX spectra of the coated surfaces before and after 3000 thermocycles. The spectra confirmed the persistence of antimicrobial elements (Ag and Cu) on the coating surfaces of the GAg and GAgCu groups following thermal aging, demonstrating the stability of these elements despite thermal cycling. While the sodium peak was present before thermal aging, it was no longer detectable after aging, indicating that sodium decreased to levels below the detection limit. The group names are indicated above each graphic. (−), before aging; (+), after aging.

The SEM images obtained from the specimens’ surfaces after thermal aging demonstrated that all coatings uniformly covered the alloy surface, with no defects or delamination observed. The corresponding EDX spectra confirmed the persistence of Ag or Cu on the coating surfaces of the antimicrobial-coated groups following thermal aging. The SEM images are shown in Figure 4, and the EDX spectra are presented in Figure 5, while the elemental composition of the coated surfaces is summarized in Table 2.

### 3.2. Mechanical Test Results

Table 3 shows the surface and mechanical test results and statistical differences among groups, in the presence of thermal cycling or not.

Surface characterization revealed clear differences between uncoated Ti6Al4V and the coated groups, both before and after thermal cycling. Surface roughness (Ra) was lowest in the G coating (0.07 µm) and increased slightly after 3000 cycles (0.10 µm), while Ti6Al4V increased from 0.10 µm to 0.14 µm (*p* < 0.05). Surface roughness results indicated that antimicrobial-coated groups had slightly lower roughness than the uncoated alloy, but statistically similar (*p* > 0.475). The roughness of the GAg and GAgCu groups was similar to each other (*p* = 0.140). After thermal aging, Ra values were increased for all groups, but the coated groups remained statistically similar to each other (*p* = 0.645) and exhibited lower Ra values than the aged Ti6Al4V alloy (*p* < 0.001).

Contact angle measurements showed that Ti6Al4V consistently exhibited the highest values (≈51°) and remained unchanged after aging, whereas all glassy coatings had significantly lower angles (≈28–31°), with a modest increase following thermal cycling (*p* < 0.001 for coating type, *p* < 0.001 for thermal cycling). Test results revealed that both coating type (*p* < 0.001) and thermal cycling (*p* < 0.001) had significant effects on surface wettability, but their interaction was not significant (*p* = 0.120). All coatings had significantly lower contact angles (more hydrophilic surface) than uncoated Ti6Al4V, before and after thermal aging (*p* < 0.05).

**Figure 6:**
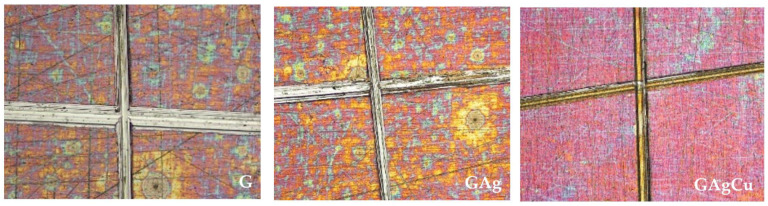
Cross-hatch adhesion test images of the coated surface obtained at ×10 magnification. No peeling or detachment was observed around the edges of the scratches, and the coating was classified as 5B according to ASTM D3359 [39], indicating excellent adhesion.

Microhardness values were higher in all coatings (240–277 HV) compared to Ti6Al4V (136–146 HV), and these values remained largely stable after aging (*p* < 0.001). Adhesion testing according to ASTM D3359 [39] confirmed excellent film adherence in all coated groups, with the highest rating (5B-meaning 0% peel-off) maintained before and after aging. Figure 6 presents the stereomicroscopic images of the coated groups after the ASTM D3359 [39] tape adhesion test application.

The critical load values revealed that GAg and GAgCu coatings exhibited better scratch resistance than the G coating, before aging. Scratch resistance was highest in Ag- and Ag/Cu-containing coatings (≈3.9 N) pre-aging. However, after aging, the GAgCu coating showed a decrease and became similar to the G coating. Otherwise, the GAg coating had higher scratch resistance, even after aging.

### 3.3. Microbial Adhesion Test Results

The three-way ANOVA results showed that the coating material, thermal aging, and the strain of the microorganism are effective factors for microbial adhesion to the groups (*p* < 0.001). Fluorometric analysis absorbance values (excitation/emission: 530/560 nm, Mean ± SD) of *Candida albicans* DSM 5817, *Porphyromonas gingivalis* ATCC 33277, and *Streptococcus sanguinis* ATCC 10556 on uncoated Ti6Al4V alloy and glassy coating groups, indicating microbial adhesion before and after aging, are presented in Table 4 and Table 5.

According to the results, antimicrobial coatings significantly lower fluorescence intensity compared to the G coating and the uncoated Ti6Al4V control group, both before and after thermal aging. When comparing pre- and post-aging conditions, microbial adhesion significantly increased after aging in all groups (*p* < 0.05), but the antimicrobial coatings maintained comparatively lower adhesion levels despite the increase. Pairwise comparisons with Bonferroni correction showed that the GAgCu glassy coating exhibited significantly lower microbial adhesion compared with the uncoated Ti6Al4V alloy, GAg, and G groups (*p* < 0.01). Before thermal aging, total microbial accumulation regardless of the microbial strain showed that GAgCu was better than GAg (*p* < 0.001). After aging, GAgCu exhibited similar adhesion with GAg, but still exhibited lower microbial adhesion than GAg for *P. gingivalis* and *S.sanguinis* strains. As shown in Table 4, compared to the GAg coating, the GAgCu coating demonstrated approximately 57% lower adhesion of *C. albicans*, 26% lower adhesion of *P. gingivalis*, and 46% lower adhesion of *S. sanguinis*. These reductions were statistically significant (*p* < 0.05), indicating that the incorporation of both silver and copper into the glassy matrix further enhanced the antibacterial and antifungal efficacy of the coating compared to silver alone. After aging, as shown in Table 5, compared to the GAg coating, the GAgCu coating demonstrated approximately 3% lower adhesion of *C. albicans* (not statistically significant), while adhesion of *P. gingivalis* and *S. sanguinis* was reduced by approximately 48% and 34%, respectively (*p* < 0.05).

The levels of microbial adhesion to the specimens varied depending on the strain. Before aging, *S. sanguinis* exhibited the highest adhesion among all tested strains. Different material surfaces also influenced microbial adhesion. In the Ti6Al4V control group, *P. gingivalis* adhered more than *C. albicans* before aging, whereas this relationship was completely reversed after aging. On aged surfaces, *C. albicans* adhesion increased markedly, reaching levels comparable to *S. sanguinis*, while *P. gingivalis* showed only a slight increase. Before aging, *C. albicans* exhibited the lowest adhesion, whereas after aging, the lowest adhesion was observed for *P. gingivalis*.

### 3.4. Cytotoxicity Test Results

#### 3.4.1. Agar Diffusion Test Results

**Figure 7:**
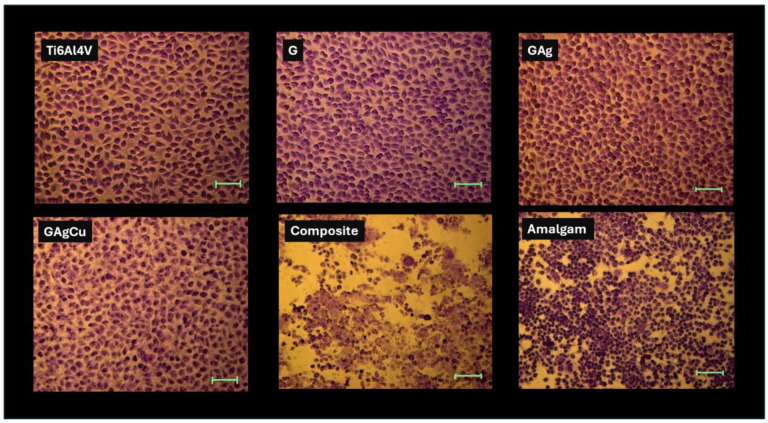
Phase-contrast microscopic images of non-aged specimens after the agar diffusion test. Scale bar: 100 µm.

The phase contrast microscopic images obtained non-aged specimens after the agar diffusion test are presented in Figure 7. As shown in the microscopic images, the Ti6Al4V, G, and GAg groups were evaluated as non-toxic. However, the GAgCu exhibited slight cytotoxicity with minimal lysis, and >90% viability was observed in the cells under and around the material. Therefore, the GAgCu group was evaluated as grade +1 toxicity following ISO 10993-5 [42]. Dental composite filling materials exhibited significant lysis and higher toxic effect (>+3 toxicity), and the amalgam filling material had severe cytotoxicity with extensive lysis and <50% cell viability (+4 toxicity).

#### 3.4.2. MTT Assay Results

**Figure 8:**
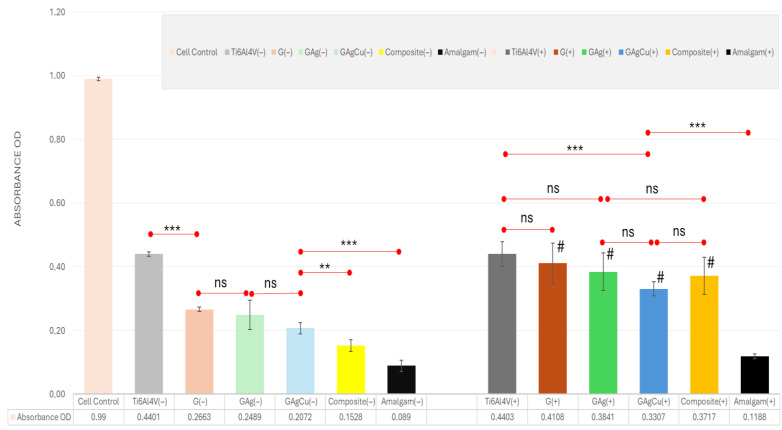
Graphical presentation of the MTT assay (absorbance OD) results of the groups. Before thermal aging, all glassy coatings exhibited lower cell viability compared with uncoated Ti6Al4V, yet remained higher than composite and amalgam. After aging, cell viability increased, with the G coating reaching a level comparable to titanium, while GAg and GAgCu showed values similar to each other and the composite, but still retained better performance than amalgam. OD, optical density; (−), before aging; (+), after aging; ** *p* < 0.01; *** *p* < 0.001; ns, not significant. # Indicates groups with significant differences after aging (*p* < 0.05).

According to MTT assay results, Ti6Al4V exhibited the highest cell viability, which was significantly greater than all other groups (*p* < 0.001), both before and after aging. Antimicrobial glassy coatings (GAg and GAgCu) showed comparable results to each other (*p* > 0.238), but were all markedly inferior to Ti6Al4V (*p* < 0.001). Composite demonstrated significantly lower viability than the coatings (*p* < 0.041), while amalgam was the least biocompatible material, presenting significantly lower values than every other group (*p* < 0.001 vs. coatings, *p* = 0.009 vs. composite). After aging, Ti6Al4V maintained the highest cell viability and was similar to the G, GAg, and composite (*p* > 0.06). In contrast, Ti6Al4V showed significantly higher values compared with GAgCu (*p* < 0.001). The antimicrobial glassy coatings exhibited similar biocompatibility to each other (*p* > 0.064) and the composite (*p* > 0.05). Amalgam again remained the most cytotoxic group, showing markedly lower values than all other groups (*p* < 0.001). Figure 8 illustrates the spectrophotometric absorbance (optical density) values obtained from aged and non-aged groups in the MTT assay, along with their statistical comparisons.

## 4. Discussion

Coating implant surfaces with antimicrobial layers is an important topic in the literature. Most research on surface modifications of dental or medical implants has focused on the implant body that integrates with bone, with the main goal of improving osseointegration. In this context, various bioactive and antimicrobial glassy materials have been developed [44,45,46,47,48,49]. The glassy coatings we developed in this study are different from previous works, both in composition and in their design specifically for abutment surfaces. Our goal was to create smooth coatings that could strongly adhere to an already smooth surface. To achieve this, the titanium substrates were machined using CNC to mimic the milled surface of abutments, producing surfaces smoother than the thresholds recommended for dental materials. Coating abutments with a glassy layer is not entirely new. Some previous studies [47,48,49] have attempted to coat abutments with glass and even make them antimicrobial. However, previous approaches relied on conventional methods, which require very high temperatures, around 900–1000 °C. A key novelty of our study is that we developed a glassy coating that can be applied at low temperatures. This approach prevents the abutment from being exposed to high temperatures, allowing for the formation of a thin, uniform glassy film on its surface.

In the present study, glassy coatings were synthesized by combining MTEOS as a functional precursor and TEOS as the source of SiO_2_. Subsequent condensation reactions promoted the in situ formation of oxide particles, resulting in the development of nanocomposite glassy systems. Sodium hydroxide was added as a Na_2_O source, serving both as a catalyst and as an agent to reduce the glassy transition temperature. The choice of 450 °C curing heat was based on literature [50], indicating that a temperature range of 400–500 °C is suitable for glassy coatings derived from MTEOS/TEOS. This is an intermediate temperature of 450 °C was selected, and since no issues were observed in the preliminary tests, this temperature was used throughout the study. Another reason for using a single temperature for all matrices was the consistency with a previous report [24], which used one temperature for different matrices. Additionally, it provides practical ease of application for potential users. Our preliminary experiments conducted on medical-grade-5 titanium alloy, commonly used for dental implant abutments, revealed that a mixture with an MTEOS/TEOS/NaOH ratio of 4/1/0.6 produced a stable glassy coating through hydrolysis and condensation reactions. This system was subsequently modified with Ag and Cu ions to impart antimicrobial properties, and its deposition onto the titanium alloy surface was optimized. The resulting coatings were evaluated in terms of mechanical durability, surface characteristics, antimicrobial performance, and cytocompatibility.

The previous report on antibacterial coatings prepared from hexadecyltrimethoxysilane and tetraethoxysilane has demonstrated that a minimum metallic silver content of 0.5% is required to achieve antibacterial efficacy against *E. coli* and *S. aureus* [51]. Therefore, in our study, the total silver content was adjusted to 1% and 2%. Increasing the concentration beyond these levels was considered likely to enhance cytotoxic effects despite the potential for improved antibacterial performance. Furthermore, as the synergistic antimicrobial effect of silver and copper combinations has been reported in the literature [52], coatings containing either silver alone or silver–copper combinations were prepared in the present study.

The roughness of the intraoral parts of the dental implants plays an important role in microbial adhesion and colonization [53]. Earlier studies showed that significantly higher microbial accumulation occurs on rough surfaces than on smooth sites [37]. However, the results of in vivo studies on implant abutments reported that a further reduction in Ra value, less than 0.2 µm, had no significant effect on the microbial composition [53]. Therefore, this value was accepted as “threshold Ra” for dental applications to prevent excessive plaque accumulation on intraoral hard materials [37]. When producing new antimicrobial coatings for dental implants, it should be considered surface roughness as an important parameter to avoid exceeding the accepted threshold Ra value, because silver and copper in the coating decrease over time as they are used by bacteria [54]. Even if the initial number of bacteria adhering to the surface is low due to the antimicrobial effect of the coating, if the coating layer has a rougher surface than the threshold value Ra = 0.2 µm, it may lead to excessive microbial accumulation over time. In a previous study [47], antimicrobial glassy coatings with silver nanoparticles for dental implant abutment had been produced via grinding and melting of soda lime glass (traditional method of glass production), but this coating had an approximately 1 µm Ra value. Since the processes in the sol–gel method are performed at the molecular level, much more homogeneous and smooth coating surfaces can be obtained in our work than in the traditional method of glass production. Previous studies [24,55] synthesized silica-based glassy coatings for metal substrates that had been used as antimicrobial agents in particulate form, such as zeolite. In our study, to prevent rough surface formation, antimicrobial agents incorporated into the glassy structure were used in ionic form. According to the results obtained from surface roughness measurements, both initial and after thermal aging, the roughness values of all coatings were below Ra 0.2 µm. In addition, all coating materials did not lead to significant changes in surface roughness when compared to the uncoated Ti6Al4V alloy. Although thermal aging caused an increase in mean surface roughness of coatings, there was no statistically significant difference between the coated groups and the uncoated alloy. All these results indicated that the glassy coatings developed in this study have suitable properties for dental usage in terms of surface roughness.

Hydrophilicity is widely recognized as a key feature for abutment and transmucosal surfaces of the dental implants, as it promotes the effective attachment of gingival fibroblasts and helps prevent apical migration of microorganisms [56,57]. The surface contact angle results indicated that the glassy coatings were more hydrophilic than the uncoated Ti6Al4V alloy. The addition of Ag or Cu to the G coating did not significantly affect surface wettability, likely because these elements are incorporated at the molecular rather than particulate level. After aging, the hydrophilicity of all coatings decreased, most likely due to the leaching of sodium ions from the surface under thermal stress, as suggested by EDX analysis. The unmodified glassy coating (G group) demonstrated a smoother and more hydrophilic surface than the Ti6Al4V alloy. While aging slightly reduced hydrophilicity, the surfaces remained hydrophilic (contact angle <90°), indicating that even without antimicrobial agents, the G coating may still offer beneficial properties for titanium abutments. Although coatings with antimicrobial agents are naturally preferred, it is important to note that their effect relies on the gradual release of active substances, which will inevitably be depleted over time. Once this occurs, the surface properties will become similar to those of the non-antimicrobial G coating. Therefore, understanding and evaluating the inherent properties of the G coating is particularly important, as it highlights its potential to maintain favorable surface characteristics even in the absence of antimicrobial activity.

Surface hardness for dental materials indicates how well a surface can endure mechanical forces such as scratching, abrasion, and deformation. Since the surface hardness of titanium and its alloys is not high, there is a risk of scratching the implant surfaces during clinical examination or cleaning the contaminated implant surface with metallic instruments used in dentistry [58,59]. According to surface hardness test results in our study, glassy systems exhibited statistically higher hardness than the Ti6Al4V alloy. This suggests that these coatings are more advantageous in clinical terms and are more resistant than Ti6Al4V surfaces. After thermal aging, there was no significant change in the hardness of the GAg and GAgCu groups. However, there is a significant reduction in the hardness of the G group. This may be explained by the resulting decondensation of the sodium ions in the glassy network. As the addition of silver and silver–copper to the system reduced the dissolution, the reduction in hardness was much less and did not make a statistically significant difference.

Another important factor affecting the clinical performance of a surface coating material is the adhesion capability to the substrate material. It is also necessary for antimicrobial coatings to have sufficient adhesion strength on implant surfaces to prevent coating delamination. In our study, according to the tape adhesion test performed in accordance with ASTM D3359 [39], all studied layers showed no disintegration and had excellent adhesion (i.e., 5B meaning 0% peel off) to the surface of Ti6Al4V substrate, even after aging. In a previous study, which used the same test standard for assessment of coating adhesion, it was reported that 4B adhesion value has good mechanical properties for dental implant applications [60]. Therefore, the obtained adhesion values in this study, even after thermal aging, were 5B, indicating that the synthesized coatings were successful and clinically acceptable in terms of adhesion. According to scratch test results, incorporating Ag and Ag/Cu into the sol-gel matrix improved the scratch resistance of the coatings. This enhancement is likely due to the catalytic activity of the metal ions during the sol-gel condensation reaction, which increases the crosslinking density of the coating network and, consequently, the coating’s scratch resistance. The addition of Ag led to a stronger adhesion of the coating to the substrate. This result shows that, by adding Ag to the system, the de-condensation resulting from the loss of sodium ions in the glassy structure can be reduced, and a better bonding to the substrate can be achieved. Furthermore, after thermal cycling, the GAg demonstrated enhanced scratch resistance, whereas the GAgCu exhibited a notable reduction in scratch resistance. This observation implies that the incorporation of Cu may affect the crosslinking stability of the coating when subjected to thermal stress. Further research is necessary to clarify the mechanisms responsible for this behavior.

The SEM analysis revealed that the glassy coatings uniformly covered the alloy surfaces, with no observable defects, cracks, holes, or delamination. The EDX findings indicate that the antimicrobial agents, Ag and Cu, remained incorporated within the coating structure even after aging. A significant decrease in sodium content was observed across all coatings following aging. This reduction is likely attributable to the ionic nature of sodium, which forms weaker bonds within the glassy matrix compared to the more stable Si–O–Si network, making it more prone to leaching under aging conditions.

Dental plaque accumulation plays a critical role in the development of peri-implantitis, and this process begins primarily with early colonizers that bind to pellicle proteins on the material surface. Among these bacteria, *S. sanguinis* stands out for its strong adhesion ability to the pellicle covering intraoral hard tissues [2,3]. Furthermore, its identification as one of the main causative agents of infective endocarditis elevates the clinical importance of this microorganism beyond oral health [61]. In our study, *S. sanguinis* showed the highest level of adhesion compared to other microorganisms, which is consistent with data in the literature. A previous study [62] has also reported that this species adheres more to titanium abutment surfaces than *P. gingivalis*. The antimicrobial effects of silver and copper against Streptococcus species and their role in reducing plaque accumulation when incorporated into dental materials have been emphasized in many studies [32,63]. When the microbial adhesion–reducing effects of the coating matrices in our study were assessed, GAg and GAgCu significantly decreased the adhesion of *S. sanguinis*, an early colonizer, in accordance with the literature. This finding suggests that these coatings may be effective in preventing plaque formation and may potentially contribute to the prevention of peri-implantitis.

*Candida albicans* is often detected in peri-implantitis cases and is known to contribute to the microbial profile of peri-implant sulcular fluid, as well as to the development of thick biofilm layers on implant surfaces [64,65]. As biofilm matures, the presence of *C. albicans* helps stabilize the microbial community and supports the plaque matrix formed by early colonizers such as streptococci. This process increases the risk of inflammation and tissue breakdown, playing a role in the progression of peri-implant disease. Another clinically relevant feature of *C. albicans* is its strong tendency to adhere to polymeric materials [66], which enables long-term colonization on denture bases and makes management of fungal infections more difficult. Therefore, strategies that reduce *C. albicans* adhesion to abutment surfaces can be considered clinically valuable. In our study, the GAgCu coating, containing both silver and copper, showed a more pronounced reduction in *C. albicans* adhesion compared with the GAg coating. Before aging, *C. albicans* adhesion levels were lower than those of *S. sanguinis*, which can be explained by the fact that early colonizers such as *S. sanguinis* can attach more quickly and in greater numbers to pellicle-coated surfaces [2,3]. We also observed a significant increase in *C. albicans* adhesion after thermal aging, which is consistent with the results of Hahnel et al. [67], who reported a marked rise in candidal adhesion following aging. However, GAgCu coating still had lower candidal adhesion when compared to the other groups. This finding suggests that using GAgCu-coated abutments may be advantageous for patients at higher risk of fungal infection, for example, those wearing implant-supported removable prostheses or resin-based hybrid prostheses.

*Porphyromonas gingivalis* is a key anaerobic pathogen in the oral cavity, strongly associated with periodontal destruction, peri-implantitis, and alveolar bone loss [68], and has more recently been linked to systemic conditions such as Alzheimer’s disease [69]. It typically colonizes soft tissue surfaces, such as the gingiva, or establishes itself as a secondary colonizer within existing microbial biofilms. In our study, when overall microbial adhesion was evaluated, *P. gingivalis* exhibited the lowest adhesion capacity among the tested species. This may be attributed to its relatively low affinity for binding to hard surfaces compared with other microorganisms. A previous study [70] also reported that *P. gingivalis* adheres less strongly to solid surfaces than *C. albicans* and *S. sanguinis*. After thermal aging, adhesion levels increased for all microorganisms; however, the most pronounced increase was observed in *C. albicans*, while *P. gingivalis* showed the least change. *C. albicans* displayed a dramatic increase after aging, reaching levels comparable to *S. sanguinis.* Since *C. albicans* is known to prefer hydrophobic surfaces, the aging-related increase in contact angle may have facilitated its enhanced adhesion. This finding suggests that thermal aging may render abutment surfaces more susceptible to fungal colonization, potentially promoting biofilm maturation under clinical conditions. By contrast, *P. gingivalis* demonstrated only a minor difference in adhesion between pre- and post-aging groups, indicating that its adhesion properties to the surface may be less sensitive to changes in surface energy compared with the other tested species.

When the antimicrobial performance of the coating matrices was evaluated, Ag- and Cu-doped coatings (GAg and GAgCu) significantly reduced microbial adhesion across all tested strains, and their effectiveness was maintained even after material aging. The G coating, which did not contain any antimicrobial agents, exhibited a similar level of microbial adhesion to that of the uncoated titanium alloy. Among the coating matrices, the GAgCu coating exhibited the greatest reduction in microbial adhesion. These findings indicate that the incorporation of silver and copper into the glassy coatings exerts a synergistic effect, resulting in enhanced antimicrobial performance against microorganisms. In the literature [71], copper oxide nanoparticles have been reported to exhibit strong bactericidal effects not only against Gram-positive but especially against Gram-negative bacteria. Vargas-Reus et al. [72] demonstrated that the combined use of silver and copper nanoparticles produced a stronger antimicrobial effect against peri-implantitis pathogens compared with either agent alone. However, these studies tested nanoparticles in isolation and not as part of a coating system. In our study, silver and copper were incorporated together into the glassy coating, and the GAgCu coating was obtained, which demonstrated the strongest antimicrobial activity against all three bacterial species tested. Owing to the dense inorganic matrix, the GAgCu group was also able to maintain its antimicrobial effectiveness even after the aging process.

When the relationship between the physical surface properties of the coatings and microbial adhesion was evaluated, surfaces with higher surface roughness and lower contact angle values exhibited an increased tendency for microbial attachment, particularly in the *S. sanguinis* group. This finding is consistent with previous studies emphasizing the critical role of surface topography in bacterial adhesion [37,73]. However, this correlation was not observed linearly in GAg and GAgCu coatings. Despite slight increases in roughness and a more hydrophilic character after aging, these coatings exhibited significantly lower microbial adhesion. This suggests that the antimicrobial efficacy of Ag^+2^ and Cu^2+^ ions has a more dominant influence on microbial eradication than surface morphology, which is in agreement with previous reports [74,75]. Therefore, although physical surface parameters partially contribute to microbial adhesion behavior, the ion-release-mediated antimicrobial activity appears to be the primary determinant of the overall microbial eradication capacity of the matrix.

Titanium and its alloys are widely used in endosseous implant applications due to their excellent biocompatibility. Abutments, however, do not come into direct contact with bone but rather interact only with the gingival tissues. For this reason, in the present study, the biocompatibility of the glassy coatings was evaluated using fibroblast cells. According to the literature, dental restorative materials in close contact with gingival tissues may exhibit mild to moderate cytotoxic effects [76]. Since abutments are positioned in a similar location and in contact with the gingiva, dental restorative materials were used as the additional control group in the biocompatibility tests to determine whether the glassy coatings are suitable for intraoral use. When considering the biocompatibility of the coating matrices, the findings revealed that the G, GAg, and GAgCu coatings were slightly more cytotoxic than the titanium alloy, but more biocompatible than restorative materials. In the pre-aging agar diffusion test, the G and GAg groups were classified as non-toxic, while the GAgCu group demonstrated a slight increase in cytotoxicity. Although copper is generally considered biocompatible at low concentrations, several studies have reported that it may induce morphological alterations and reduce cellular proliferation depending on concentration and exposure time [77,78]. In our study, when GAg and GAgCu were compared, a slight reduction in cell viability was observed in the GAgCu group due to copper, although this difference did not reach statistical significance. Considering that the GAgCu coating provided a more pronounced reduction in microbial adhesion, particularly against *C. albicans*, this minor cytotoxicity increase can be regarded as clinically acceptable. It can be suggested that the coatings exhibit a level of biocompatibility sufficient for application on abutment surfaces.

After aging, all coating groups showed an increase in cell viability. This finding is likely related to the release and subsequent reduction of cytotoxic components within the material during aging, which is consistent with similar reports in the literature [79,80]. In contrast, the amalgam group exhibited no significant change after aging, maintaining its high level of cytotoxicity. Previous studies on glassy coatings have also reported no adverse effects on L929 cells [81,82], and our results support these findings, demonstrating greater biocompatibility compared with amalgam. In conclusion, GAg and GAgCu exhibited slightly lower cell viability than titanium; however, their overall biocompatibility remained within a clinically acceptable range. In particular, the GAgCu coating, despite a minor increase in cytotoxicity, showed strong antimicrobial effects, indicating promising potential for application on abutment surfaces. Nevertheless, in vivo studies are essential before clinical translation can be confirmed.

Based on the results obtained from experimental tests, the null hypothesis of the study, which proposed that the glassy coatings did not cause any change in the mechanical, surface, microbial adhesion, and cell viability properties of the Ti6Al4V surface, was rejected.

Considering the clinical applicability of glassy coatings, the findings of this study suggest that GAg coatings may be more advantageous in areas where mechanical durability is essential and where abrasive forces such as toothbrushing are expected. On the other hand, in cases where infection control is a more critical factor, particularly in patients at high risk of peri-implantitis, the use of GAgCu coatings would be more beneficial. Further studies are needed to evaluate their performance under in vivo conditions. In our study, the developed coatings maintained their stability after 3000 thermal cycles, simulating approximately 3.5 months of clinical use. This finding indicates that these coatings could be safely applied to healing abutments or temporary abutments. However, since permanent prosthetic abutments are intended for long-term intraoral use, their long-term resistance to aging conditions should be investigated in future research.

This study has certain limitations. The findings were obtained exclusively under in vitro conditions and therefore require validation through comprehensive in vivo studies that better reflect long-term clinical situations. Additionally, only a single, short-term aging protocol was employed, and neither alternative coating techniques nor multilayer applications were investigated. Antimicrobial activity was tested against only three microbial strains, which does not fully capture the complexity of the oral microbiota. The antimicrobial agents used were also limited to silver and silver–copper combinations. Nevertheless, the results demonstrate the feasibility of the method and provide a foundation for future clinical and laboratory research.

## 5. Conclusions

Within the limitations of this study, it can be concluded that sol–gel-derived silica-based glassy coatings synthesized in this study can be applied to medical-grade Ti6Al4V alloy surfaces. Using Ag and Cu in ionic form for sol–gel-derived glassy silica structure, both have low roughness coating surfaces, and thermal cycle-resistant coatings can be obtained. Especially, the addition of Ag to the glassy system leads to an improvement in the mechanical performance of the coating after thermal aging, compared to pure glassy coating. Because of their superior surface and mechanical properties, strong adhesion to substrate, adequate biocompatibility, and antimicrobial effectiveness even after thermal cycling, these coatings can be preferred on titanium-based dental implant abutments for antimicrobial purposes.

## Figures and Tables

**Table 1:** The compositions of the synthesized glassy coating solutions in this study.

	G	GAg	GAgCu
**Glassy matrix solution**	26.78 g	25 g	25 g
**Ag-GF20 complex solution**	-	4.17 g	2.06 g
**Cu solution**	-	-	2.06 g
**Butyl glycol (BG)**	2.5 g	2.5 g	2.41 g
**Ethanol (EtOH)**	21.88 g	16.89 g	16.60 g

**G:** Glassy coating, **GAg:** Glassy coating with 2% Ag, **GAgCu**: Glassy coating with 1% Ag and 1% Cu.

**Table 2:** Elemental composition (wt%) of the coated surfaces before and after 3000 thermocycles.

GROUP	Thermal Aging	C%	Na%	O%	Si%	Ti%	Al%	V%	Ag%	Cu%	N%
**G**	None	11.56	4.07	74.62	37.74	21.19	1.21	1.17	-	-	-
	3000 cycles	4.99	0.01 *	31.25	12.50	28.69	1.51	1.47	-	-	-
**GAg**	None	9.22	4.38	50.97	10.57	21.25	1.08	1.20	8.79	-	-
	3000 cycles	5.80	0.01 *	50.98	7.24	41.47	2.41	1.54	7.66	-	-
**GAgCu**	None	6.74	5.70	52.78	12.67	21.15	2.00	1.06	1.21	0.30	-
	3000 cycles	4.72	0.01 *	49.40	7.13	35.48	1.17	1.00	1.17	0.20	-

* 0.01: Not detected in the EDX analysis after aging; considered to have decreased to levels undetectable by EDX.

**Table 3:** Mechanical and surface analyses test results of the experimental groups (Mean ± SD).

	Thermal Cycling	Surface Roughness Ra (µm)	Surface Contact Angle (°)	Surface Hardness (HV)	Scratch Resistance (N)	ASTM D3359 [39]
**Ti6Al4V**	None	0.10 ± 0.01 ^a^	51.6 ± 0.5 ^a^	136 ± 5.3 ^a^	-	-
	3000 cycles	0.14 ± 0.03 ^b^	51.7 ± 0.6 ^a^	146 ± 10.3 ^a^	-	-
**G**	None	0.07 ± 0.017 ^c^	28.0 ± 1.6 ^b^	262 ± 35.7 ^b^	2.81 ± 0.89 ^a^	5B
	3000 cycles	0.10 ± 0.039 ^a^	35.4 ± 1.9 ^c^	240 ± 52.9 ^b^	2.24 ± 0.14 ^a^	5B
**GAg**	None	0.09 ± 0.038 ^a^	29.6 ± 2.7 ^b^	277 ± 40.5 ^b^	3.90 ± 0.80 ^b^	5B
	3000 cycles	0.12 ± 0.042 ^ab^	33.9 ± 1.4 ^c^	261 ± 47.7 ^b^	4.56 ± 0.39 ^b^	5B
**GAgCu**	None	0.08 ± 0.017 ^ac^	30.5 ± 1.6 ^b^	252 ± 51.3 ^b^	3.95 ± 0.55 ^b^	5B
	3000 cycles	0.11 ± 0.027 ^a^	33.3 ± 2.2 ^c^	272 ± 55.4 ^b^	2.72 ± 0.53 ^a^	5B

Different superscript letters indicate statistical differences in a column (*p* < 0.05). SD: Standard deviation. HV: Hardness Vickers. N: Newton.

**Table 4:** Fluorometric Absorbance Values (530/560 nm, Mean ± SD) indicating microbial adhesion before aging.

Groups	*C. albicans* DSM 5817	*P. gingivalis* ATCC 33277	*S. sanguinis* ATCC 10556
**Ti6Al4V**	4.8 ± 0.98 ^a,A^	7.7 ± 0.9 ^a,B^	27.3 ± 0.48 ^a,C^
**G**	4.5 ± 1.02 ^a,A^	9.7 ± 1.4 ^b,B^	28.8 ± 12.26 ^a,C^
**GAg**	2.3 ± 0.48 ^b,A^	3.1 ± 1.04 ^c,A^	6.5 ± 1.42 ^b, B^
**GAgCu**	1.0 ± 0,12 ^c,A^	2.3 ± 0.29 ^d,B^	3.5 ± 0.38 ^c,B^

Different superscript small letters within the same column and capital letters within the same line indicate statistically significant differences (*p* < 0.05).

**Table 5:** Fluorometric Absorbance Values (530/560 nm, Mean ± SD) indicating microbial adhesion after aging.

Groups	*C. albicans* DSM 5817	*P. gingivalis* ATCC 33277	*S. sanguinis* ATCC 10556
**Ti6Al4V**	157.9 ± 13.2 ^a,A^	9.8 ± 0.84 ^a,B^	166.9 ± 25.98 ^a,A^
**G**	131.6 ± 7.6 ^a,A^	13.4 ± 1.12 ^b,B^	185.0 ± 13.90 ^a,C^
**GAg**	111.6 ± 8.1 ^b,A^	6.7 ± 0.86 ^c,B^	137.6 ± 14.57 ^b,C^
**GAgCu**	108.5 ± 10.9 ^b,A^	3.5 ± 0.29 ^d,B^	90.2 ± 16.55 ^c,C^

Different superscript small letters within the same column and capital letters within the same line indicate statistically significant differences (*p* < 0.05).

## Data Availability

The original contributions presented in this study are included in the article. Further inquiries can be directed to the corresponding author.

## Supplementary Materials

The following supporting information can be downloaded at: https://www.mdpi.com/article/10.3390/gels11110882/s1, Table S1: Preliminary Optimization Trials for Antimicrobial Coating Combinations.

## Abbreviations

The following abbreviations are used in this manuscript:

Abbreviations	Full Form
MTEOS	Methyltriethoxysilane
TEOS	Tetraethoxysilane
NaOH	Sodium hydroxide
Al_2_O_3_	Aluminum oxide
EtOH	Ethanol
BG	Butyl glycol
Ag	Silver
Cu	Copper
DNA	Deoxyribonucleic acid
ASTM	American Society for Testing and Materials
ISO	International Organization for Standardization
RCF	Relative centrifugal force
GAM	Gifu Anaerobic Medium
MTT	3-(4,5-dimethylthiazol-2-yl)-2,5-diphenyltetrazolium bromide
DMEM	Dulbecco’s Modified Eagle Medium
SEM	Scanning Electron Microscopy
EDX	Energy Dispersive X-ray Spectroscopy
ATCC	American Type Culture Collection
DSM	Deutsche Sammlung von Mikroorganismen und Zellkulturen
