# Supplementary material for: Antimicrobial Sol–Gel Glassy Surfaces for Modification of Dental Implant Abutments to Reduce Microbial Adhesion
Source: Gels. 2025 Nov 3;11(11):882. doi: 10.3390/gels11110882 (PMC12652457; doi:10.3390/gels11110882)
Supplement: Supplementary file 1 [file gels-11-00882-s001.zip › gels-3913209-supplementary.pdf]

Table S1. Preliminary Optimization Trials for Antimicrobial Coating Combinations.

Experimental trials	Composition (in solid)	System	GM (g)	GM (Hyd) in PM (g)	GM (Hyd) in IPA (g)	GM (Hyd) in EtOH (g)	Ag-GF20 (g)	Cu-GF20 (g)	1.75 % Ag (g)	1.75 % Cu (g)	H_2O (g)	PM (g)	IPA (g)	EtOH (g)	RESULTS
1	1% Ag + 1% Cu	GAgCu	-	-	-	10.00	0.84	-	-	0.84	-	-	-	2.9	Acceptable
2	1% Ag + 1% Cu	GAgCu	-	-	-	10.00	0.84	0.84	-	-	-	-	-	2.9	X
3	2% Ag	GAg	-	-	-	10.00	1.67	-	-	-	-	-	-	2.9	Acceptable
4	2% Ag	GAg	-	-	10.00	-	1.67	-	-	-	-	-	-	-	X
5	1% Ag + 1% Cu	GAgCu	-	10.00	-	-	-	-	0.84	0.84	-	-	-	2.9	X
6	2% Ag	GAg	-	10.00	-	-	-	-	1.67	-	-	-	-	2.9	X
7	1% Ag + 1% Cu	GAgCu	-	10.00	-	-	0.84	0.84	-	-	-	-	-	-	X
8	2% Ag	GAg	-	10.00	-	-	1.67	-	-	-	-	-	-	-	X
9	1% Ag + 1% Cu	GAgCu	19.02	-	-	-	4.18	4.21	-	-	2.05	28.95	-	-	X
10	2% Ag	GAg	19.00	-	-	-	8.35	-	-	-	2.05	28.95	-	-	X

GM: Glassy matrix; **Hyd:** Hydrolized; **PM:** Propylene Glycol Monomethyl Ether; **IPA:** Isopropyl alcohol; **EtOH:** Ethanol; **g:** Gram; **X:** Unacceptable.
